# Semaphorin3A: A Potential Therapeutic Tool for Lupus Nephritis

**DOI:** 10.3389/fimmu.2018.00634

**Published:** 2018-04-04

**Authors:** Jacob Bejar, Ofra Kessler, Adi D. Sabag, Edmond Sabo, Ofer Ben Itzhak, Gera Neufeld, Zahava Vadasz

**Affiliations:** ^1^The Department of Pathology, Bnai-Zion Medical Center, Haifa, Israel; ^2^The Bruce Rappaport Medical School, Technion – Israel Institute of Technology, Haifa, Israel; ^3^The Division of Allergy and Clinical Immunology, Bnai-Zion Medical Center, Haifa, Israel; ^4^The Department of Pathology, RAMBAM Medical Center, Haifa, Israel

**Keywords:** semaphorin3A, NZB mice model, immune regulation, innate immunity, lupus nephritis

## Abstract

**Background:**

The immune regulatory properties of semaphorin3A (sema3A) (both innate and adaptive) are well established in many *in vitro* studies. The injection of sema3A into a mice model of rheumatoid arthritis was proven to be highly beneficial, both in attenuating clinical symptoms and in decreasing inflammatory mechanisms.

**Objectives:**

This study was designed in order to assess the possible therapeutic benefits of sema3A following its injection into female NZB/W mice.

**Methods:**

Forty-eight NZB/W mice were recruited for this study. Thirty mice were treated as a “prevention group” and 18 were used as a “treatment group.” Eight-week-old mice were acclimated and then divided into the two abovementioned groups.

**Results:**

The injection of sema3A into young mice (at week 12) before the onset of disease (the prevention group) delayed the appearance of proteinuria. Here, the median time to severe proteinuria was 110 days, 95% CI: 88–131. However, in mice in which the empty vector was injected, the median time to severe proteinuria was 63 days, 95% CI: 0–139. sema3A treatment, significantly reduced renal damage, namely, it prevented the deposition of immune complexes in the glomeruli. When sema3A was injected at the onset of proteinuria (the treatment group), aiming to treat rather than to prevent disease in these mice, survival was increased and the deterioration of proteinuria was delayed.

**Conclusion:**

Semaphorin3A is highly beneficial in reducing lupus nephritis in NZB/W mice. It delays the appearance and deterioration of proteinuria, and increases the survival rates in these mice. The regulatory mechanisms of sema3A involve both innate and adaptive immune responses. Further studies will establish the idea of applying sema3A in the treatment of lupus nephritis.

## Introduction

Systemic lupus erythematosus (SLE) is a multi-system autoimmune disease, which involves the skin, synovia, kidneys, and the brain. Long-lasting organ damage mainly, the kidneys, is associated with a high rate of morbidity and mortality. Standard therapy (steroids and immune-suppressive drugs) though beneficial and improving survival, is associated with frequent and sometimes severe side effects. Therefore, safe and better focused therapies are continuously being developed. Of these, belimumab (anti-B cell activating factor) is considered to be beneficial in milder cases of SLE (1); however, more therapies are definitely required. In this respect, the possibility of targeting regulatory cells or regulatory molecules aiming to reduce inflammation and restore self-tolerance have recently been suggested to be a beneficial strategy in the field of autoimmunity. Semaphorins are widely reported to be involved in the regulation of inflammatory immune responses. Special attention has been given to semaphorin3A (sema3A) since it is valued as one of the important regulators in suppressing immune-mediated inflammation (2). The expression of sema3A and its operative receptors, namely, neuropilin-1 (NP-1), NP-2, and plexins were reported to be increased on differentiating macrophages as well as on T regulatory cells, thereby resulting in the inhibition of T cell proliferation and pro-inflammatory cytokine secretion (3, 4). Recently, we reported that sema3A was highly expressed on B regulatory cells (CD19+CD25highCD1dhigh) suggesting that it was a possible marker for this subset of cells (5). Assuming that sema3A is involved in the pathogenesis of SLE, we designed a study where the serum level of sema3A was analyzed. Low sema3A levels were found to be negatively correlated with SLE disease activity, renal involvement, and the detection of specific autoantibodies. In this study, we were able to demonstrate increased sema3A expression on B regulatory (B reg) cells, namely, CD19+CD25high CD1dhigh. As expected, the expression of sema3A on B reg cells was significantly lower in SLE patients when compared to those in healthy individuals, thereby suggesting that this finding is partially responsible for B cell auto-reactivity in SLE (6). The overexpression of TLR-9 and increased production of pro-inflammatory cytokines such as IL-6 and IFNs is highly typical for autoreactive B cells in SLE. In many studies, these markers were reported to be positively associated with SLE disease activity as well as with increased titers of anti-dsDNA antibodies. Since it was considered to be a potent immune regulator, sema3A was cocultured with activated B cells, isolated from SLE patients, with the goal of evaluating its ability to lower TLR-9 expression. The addition of sema3A to activated B cells in culture downregulated TLR-9 expression, which raised the possibility of applying sema3A as a therapeutic option for SLE treatment (7). B-cell overactivity was also shown to be also regulated by the expression of CD72 on B cells. Following the ligation of CD72, suppressive signals are induced and B cell receptor positive signaling is downregulated, maintaining by this, self-tolerance. Keeping this in mind, we conducted a study, which sought to compare the expression of CD72 on activated B cells from SLE patients with that from healthy individuals. The expression of CD72 on B cells from SLE patients was significantly lower when compared to that in the controls. Decreased CD72 expression was inversely correlated with SLE disease activity, specifically with lupus nephritis, with high titers of anti-dsDNA antibodies and with low levels of complement (8). Here again, when purified B cells were co-cultured with recombinant sema3A, we noticed a significant upregulation of CD72 in both the normal controls and SLE patients (though they were lower than in the normal controls), suggesting its beneficial usage in many autoimmune diseases (9). The expression of sema3A was also assessed in the glomeruli and tubuli of suffering from lupus nephritis and found to be in inverse correlation with renal function assays. These findings suggested that sema3A is involved in lupus glomerulonephritis and could have a protective role (10). Taking into account all of the above factors, we designed this *in vivo* study with the aim of assessing the beneficial effect of injecting sema3A into female NZB/W mice.

Our results will show that sema3A has both therapeutic and preventive properties.

## Materials and Methods

### Mice Strains

Forty-eight female NZB/W mice were recruited for this study. Thirty mice were studied as a “prevention group” and 18 mice as a “treatment group.” Eight-week-old mice were acclimated and then were divided into the two above mentioned groups. The study was approved by the Israeli Ethical Committee for designing studies on animal models. The study was given the approval number of IL-15-12-360.

### Study Design

#### Prevention Group

Fifteen mice were injected with a recombinant human sema3A containing plasmid and another 15 were injected with empty vector cDNA and followed as a control group. Both groups were injected at the age of 12 weeks (before the appearance of proteinuria), every 3 weeks, for a total of three injections. They were followed until proteinuria appeared and when proteinuria persisted in the range of +3 (>300 mg/ml) they were sacrificed.

#### Treatment Group

Eight mice were injected with a recombinant human sema3A plasmid and eight were injected with empty vector cDNA and used as a control group. In this case, mice were injected when proteinuria was +2 (>100 mg/ml) (around 25–27 weeks of age) every 3 weeks, for a total of three injections. They were followed for the extent of proteinuria, weight loss, and their natural survival rate until they died.

### Expression Plasmids

The following specific primers were used to construct an expression vector cDNA containing human sema3A: 5′-aacgggggcttttcatcc 3′-cccttctcacatcactcatgct. The sema3A cDNA was cloned from HUVEC (human umbilical vein endothelial cells) mRNA using RT-PCR, following sub-cloning into the NSPI-CMV-myc-his lentiviral expression vector. A FLAG epitope tag was added upstream to the stop codon of sema3A as described (11).

### Hydroporation Method of Injection

Fifty micrograms of cDNA (recombinant human sema3A or empty vector) was injected in 2 ml of PBS per mouse. The injection was performed rapidly (during 5–7 s, as was described earlier) (12). In brief, a rapid injection of a large volume (above 10% of body weight) of solution (with or without any substance dissolved in it) *via* the tail vein can cause the accretion of the injected solution in the inferior vena cava. This is caused by the injection protocol of a large volume that exceeds the pump capacity of the mouse’s heart. As a result of this failure, high pressure develops in the venous region, and this causes a retrograde movement of the solution into the liver. The reason for this retrograde movement is the direct vascular connection of the liver to the inferior vena cava. As a result of this, the pore sizes of the liver fenestrae are enlarged and the membrane of the hepatocytes is permeabilized, allowing for the delivery of the injected material into the hepatocytes and over time trapping it inside. This method of hydroporation allows hepatocytes to become a source for the production of the injected recombinant protein of interest, and its gradual release into the blood’s circulation. Serum levels of human sema3A were analyzed in all mice after 4 weeks of injection and were found detectable when this was compared to mice in which empty vector was injected (data not shown). Therefore, all of the mice were injected every 3 weeks for a total three injections.

### Immunohistochemistry

In the prevention group, mice were sacrificed when proteinuria was +3 (above 300 mg/24 h). Formalin-Fixed paraffin-embedded kidney biopsies were subjected to hematoxylin–eosin [Periodic Acid-Schiff (PAS)—for evaluation of polysaccharides (especially glycogen), neutral mucus substances (glycoproteins, glycolipids, and neutral mucins), and tissue basement membranes]. We also stained kidneys with silver staining (this staining is used for highlighting the basement membrane of the glomerulus in the kidney. The periodic acid oxidizes the carbohydrate components of the basement membrane that produce aldehydes. The released aldehydes reduce the silver to visible metallic silver). The extent of glomeruli and tubule inflammation was assessed by two expert pathologists.

### Immunofluorescence

Frozen kidney biopsies were additionally assessed by immunofluorescence for the detection of IgG and C3 deposits in the glomeruli of scarified mice. The extent and intensity of these deposits was determined by two expert pathologists. The intensity and extent of staining was scored as “+3” as the highest score of immune-complex deposition and “0” as negative score.

### Statistical Methods

Survival and time to severe proteinuria progression analysis was performed using the Kaplan–Meier Analysis Curve. The difference between groups was tested using the Log-Rank test. A *p*-value of 0.05 or less was considered to be statistically significance.

## Results

### The Prevention Group

#### Proteinuria

**Figure 1:**
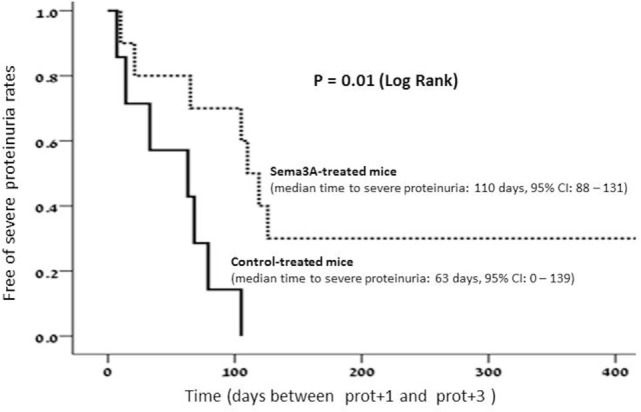
In semaphorin3A (sema3A)-treated mice, the median time to severe proteinuria was 110 days, 95% CI: 88–131. Whereas in control-treated mice, the median time to severe proteinuria was 63 days, 95%, CI: 0–139.

All mice were injected at the age of 12 weeks, before the appearance of proteinuria. Proteinuria was assessed every 4 days in all mice and was cataloged as follows: in the control sub-group, proteinuria of +1 to +2 was noticed in all mice between weeks 19 and 20. In two mice, it approached +3 (>300 mg/ml) at week 22, and in another two mice, this was documented at week 25. In six mice, proteinuria was measured +3 at weeks 30–32, and in the last five mice, it reached +3 at week 35 of age. In this group (treated with empty vector), the median time to severe proteinuria was 63 days, 95% CI: 0–139. In contrast to this, in mice in which human sema3A cDNA was injected, the time to the appearance of proteinuria was significantly longer: in the first mouse, it approached +3 (>300 mg/ml) only at week 28, and in another three mice, at week 30 of age. In four mice, proteinuria of +3 developed at 31–33 weeks of age, and in four mice, only at weeks 35–38. The remaining mice were scarified at week 40 without the appearance of any proteinuria. In this group, the median time to severe proteinuria was 110 days, 95% CI: 88–131 (Figure 1).

#### Renal Histopathology

**Figure 2:**
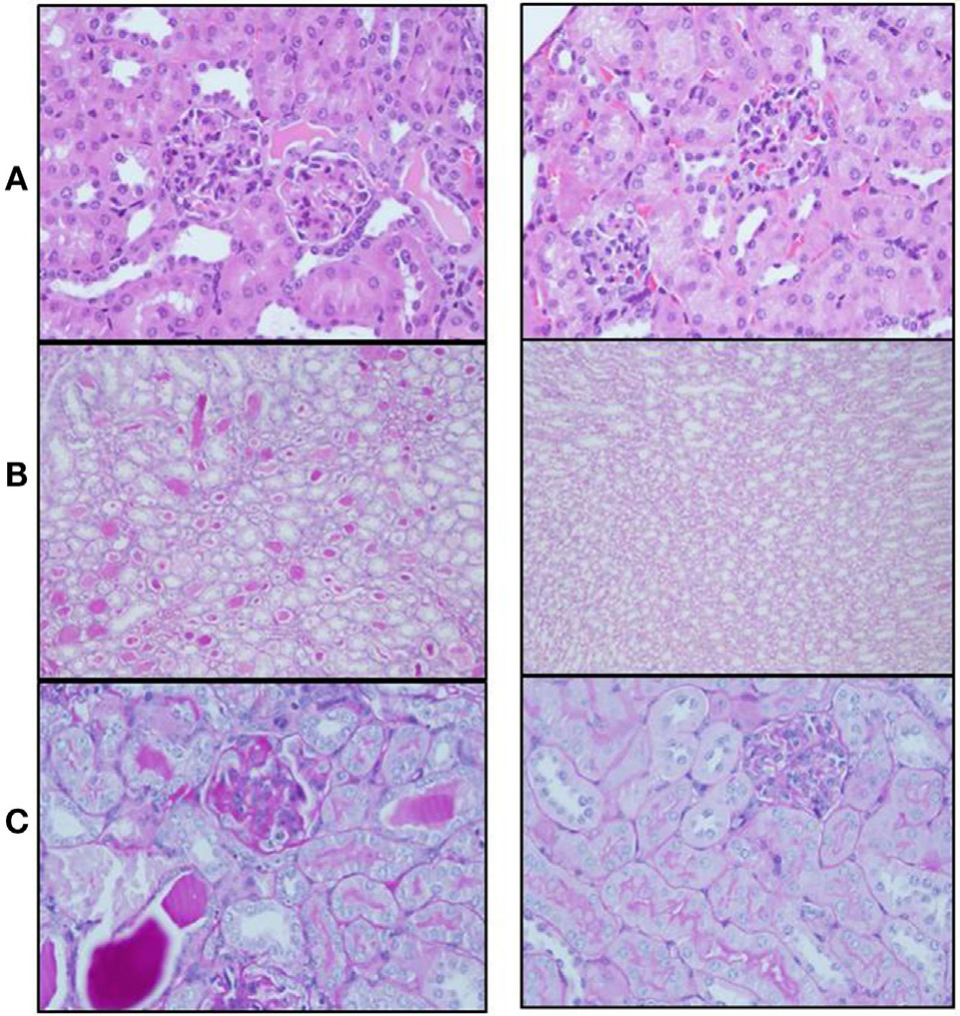
**(A)** Hematoxylin–eosin staining shows paucity of blood capillaries and diffuse mesangial proliferation in control-treated mice (left panel). These findings were minimal in semaphorin3A (sema3A)-treated mice (right panel). **(B)** PAS staining demonstrates profound glycoprotein deposits in the tubuli of control-treated mice (left panel) and minimal deposits in sema3A-treated mice (right panel). **(C)** Increased glycoprotein deposits are seen in the mesangium of control-treated mice (left panel) and minimal in sema3A-treated mice (right panel).

**Figure 3:**
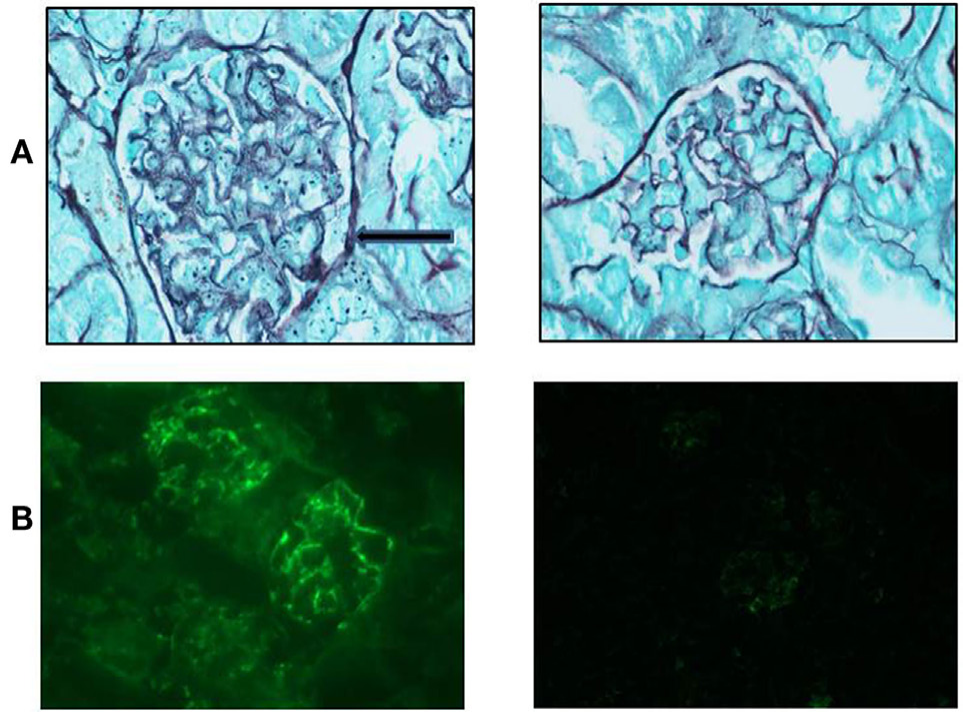
**(A)** Silver staining shows a typical railway pattern in the glomeruli of control-treated mice (left panel, black arrow). Semaphorin3A (sema3A)-treated mice remained protected with minimal glomerular damage (right panel). **(B)** Increased IgG deposition is significant in the glomeruli of control-treated mice (left panel) and is minimal in sema3A-treated mice (*p* < 0.01).

Mice from both groups (sema3A-treated and control-treated) were scarified when proteinuria reached +3 and renal biopsies were assessed: mice in which empty vector was injected, developed severe inflammation in both the interstitium and in glomeruli (see hematoxylin–eosin staining, Figure 2). The damage was noticed as paucity of blood capillaries and diffused mesangial proliferation (left panel). In sema3A-treated mice, these findings were minimal (right panel). In control-treated mice, profound glycoprotein deposits in both the mesangium and the tubuli were demonstrated using PAS staining (Figures 2B,C, left panels). This deposition was minimal in sema3A-treated mice (Figures 2B,C, right panels). When silver staining was used, a typical railway pattern was demonstrated in the glomeruli of control-treated mice (highly characteristic of lupus nephritis) (Figure 3A, left panel). In contrast, all treated mice in which sema3A was injected remained protected with minimal glomerular inflammation (Figure 3A, right panel).

#### Renal Immunofluorescence

The glomerular deposition of immunoglobulin’s (IgG) was evaluated using immunofluorescence techniques. Increased IgG deposition was significant in control-treated mice (Figure 3B, left panel). However, in sema3A-treated mice, IgG deposition was minimal (Figure 3B, right panel) (*p* < 0.01). We similarly assessed C3 deposition and found it increased in control-treated mice when compared to that of sema3A-treated mice. In this case, we could not show statistical significance due to the small number of studied mice.

### Treatment Group

#### Proteinuria

The rate of proteinuria progression from +2 to +3 was notably rapid in mice in which empty vector was injected. The time it took to progress to severe proteinuria in this group of mice was 10.2 ± 2.3 days. However, the deterioration to a proteinuria of +3 was much slower in mice in which sema3A was injected and was 18.3 ± 3.4 days (*p* < 0.2). Though indeed slower but did not reach statistical significance due to the small number of studied mice.

#### Survival

**Figure 4:**
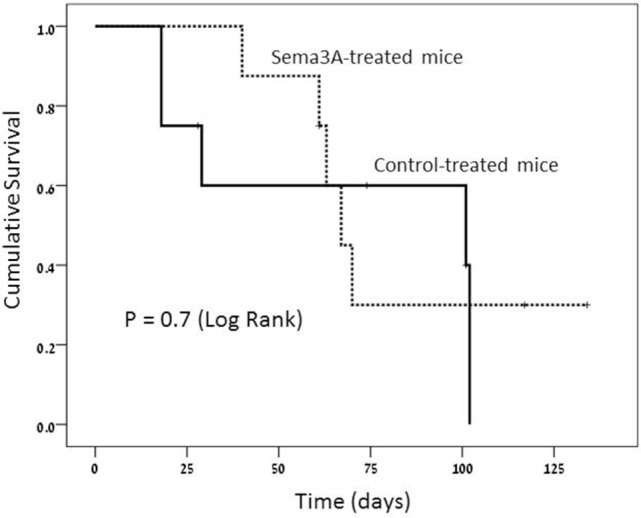
Survival rate is much longer in semaphorin3A (sema3A)-treated mice and shorter in control-treated mice.

The survival rate in the sema3A-treated vs control (empty-vector) treated mice was higher. At the end of the study, 3 out of the 8 (30%) sema3A-treated mice were still alive, whereas all the eight control-treated mice died (log rank-0.7). Though impressive, the small number of studied mice did not allow statistical significance of this difference (see Figure 4).

## Discussion

This is the first study where sema3A is demonstrated to be highly efficient in both treating and preventing glomerular damage in a SLE-mice model. When sema3A was preventively injected into NZB/W mice, they remained free of proteinuria significantly longer than control mice into which empty vector were injected. When sema3A was injected at onset of proteinuria (treatment regimen), the deterioration to severe proteinuria was significantly delayed. In both preventive and treatment regimens, the survival rate was also higher in sema3A treatment mice. By injecting sema3A into NZB/W mice, glomerular and tubular damage were notably protected, in contrast with the control mice in which glomerular inflammation and tubular damage developed quickly, leading to their short survival rate. Finally, increased immune complex deposition, mainly the deposition of IgG and C3 in the glomeruli was found in almost all control (empty-vector treated) mice. However, this deposition was minimal in sema3A-treated mice.

By injecting sema3A to NZB/W mice, glomerular inflammation and immune complex deposition were significantly decreased and the survival of these mice was prolonged. The regulatory/anti-inflammatory effects of sema3A are provided by mechanisms involving both the innate and adaptive immune responses. Aiming to evaluate the effect of sema3A on innate immune responses, dendritic cells (DCs) were incubated with sema3A-rich supernatants. Following their exposure to sema3A, MHC and CD40 expression as well as the production of IL-12 were all downregulated. The suppressive effect of sema3A was also demonstrated by the decreased capability of DCs to activate antigen-specific T cells and the secretion of IFN-γ and IL-2 (13). sema3A has been shown to influence murine DCs migration by signaling through the NP-1/plexin-A1 axis. In this respect, sema3A was further reported to influence human DC migration. By binding DCs, sema3A leads to the reorganization of actin filaments at the plasma membrane, increasing by that their cell deformability and altering their activity in the absence or presence of chemokine CCL19 (3). The role of sema3aA in controlling the function of monocyte-derived macrophages was also assessed. Here, the expression of NP-1, NP-2, and plexin A1 and A2, all of which are receptors for sema3A, were found to be significantly increased during the differentiation and activation of monocyte-derived macrophages, in association with the increased surface binding of sema3A during M2 differentiation. In addition, sema3A was able to induce apoptosis of monocyte-derived macrophages, thereby suggesting that sema3A plays a role in inducing apoptosis of activated macrophages and the modulation of innate inflammation conditions (14). One of the many molecular mechanisms by which sema3A provides its anti-inflammatory effects is by altering TCR-induced proliferation and early signal transduction responses such as ZAP-70 or focal adhesion kinase phosphorylation, resulting in a delayed negative feedback loop and the inhibition of DC-induced T cell proliferation (15). The abovementioned studies support the notion of sema3A being a regulator of innate immune responses. Obviously, these data was more than encouraging and further steps were taken and sema3A was investigated in many *in vivo* models. One of these was assessing the beneficial therapeutic effect of sema3A in a mouse model of collagen-induced arthritis. Following the injection of plasmid DNA encoding sema3A into these mice, disease severity and articular damage were significantly reduced when compared to mice injected with empty plasmid. sema3A treatment reduced the titers of anti-collagen IgG antibodies and the release of pro-inflammatory cytokines such as IL-17 and IFN-γ. In addition, sema3A treatment increased the serum level of the anti-inflammatory cytokine IL-10. In respect to this, the expression of sema3A and NP-1 on Treg cells of RA patients (appreciated as a source of IL-10) was found to be decreased. In this case, the co-culture of T-cells with sema3A induced the expression of CD4+NP-1+ T cells and increased IL-10 expression, suggesting that sema3A could be highly beneficial in treating RA (16). The expression of sema3A on synovial tissues of RA patients was also investigated, in relation with RA disease activity and synovial histological features. Human synovial tissues from established RA patients and patients suffering from osteoarthritis (OA) were assessed for sema3A, VEGF-A, and NP-1mRNA expression. Protein expression of sema3A was decreased in RA tissues when compared to that of OA, when correlated with the extent of synovial inflammation, namely, with the extent of lymphocyte infiltration (*R* = 0.50; *p* = 0.004) (17). Decreased expression/production of sema3A in the skin of atopic dermatitis as well as in psoriatic patients was found to be associated with itch and disease severity. sema3A replacement was reported to normalize the hyper-innervation in atopic dermatitis, resulting in suppression of itching suggesting sema3A to be a potential therapeutic strategy in a wide spectrum of immune-mediated inflammatory diseases (18–20).

## Conclusion

Semaphorin3A is a unique regulator of both early and late innate and adaptive immune responses. We demonstrated the high beneficial effect of sema3A in ameliorating lupus nephritis in NZB mice by delaying the appearance and deterioration of proteinuria and increasing the survival rate. Future efforts should focus on establishing *in vivo* mechanisms by which sema3A provides its beneficial effect. In addition, the above data should be supported in a bigger cohort of mice and making sema3A suitable and safe for use in SLE patients and other autoimmune diseases.

## Author Contributions

ZV conducted this study, wrote the MS, and contributed to the analysis of the results. OK, AS, and GN contributed to the semaphorin3A reagents to this study and assisted to the writing of this MS. ES contributed to the analysis of the pathology results and statistical analysis of results. JB contributed to the analysis of the pathology results. OI contributed to the analysis of the pathology results and immunofluorescence of kidneys.

## Conflict of Interest Statement

The authors declare that the research was conducted in the absence of any commercial or financial relationships that could be construed as a potential conflict of interest.
